# Population-based change-point detection for the identification of homozygosity islands

**DOI:** 10.1093/bioinformatics/btad170

**Published:** 2023-04-11

**Authors:** Lucas Prates, Renan B Lemes, Tábita Hünemeier, Florencia Leonardi

**Affiliations:** Institute of Mathematics and Statistics, University of São Paulo, São Paulo, Brazil; Institute of Biological Sciences, University of São Paulo, São Paulo, Brazil; Institute of Biological Sciences, University of São Paulo, São Paulo, Brazil; Institut de Biologia Evolutiva, Universitat Pompeu Fabra, Barcelona, Spain; Institute of Mathematics and Statistics, University of São Paulo, São Paulo, Brazil

## Abstract

**Motivation:**

This work is motivated by the problem of identifying homozygosity islands on the genome of individuals in a population. Our method directly tackles the issue of identification of the homozygosity islands at the population level, without the need of analysing single individuals and then combine the results, as is made nowadays in *state-of-the-art* approaches.

**Results:**

We propose regularized offline change-point methods to detect changes in the parameters of a multidimensional distribution when we have several aligned, independent samples of fixed resolution. We present a penalized maximum likelihood approach that can be efficiently computed by a dynamic programming algorithm or approximated by a fast binary segmentation algorithm. Both estimators are shown to converge almost surely to the set of change-points without the need of specifying *a priori* the number of change-points. In simulation, we observed similar performances from the exact and greedy estimators. Moreover, we provide a new methodology for the selection of the regularization constant which has the advantage of being automatic, consistent, and less prone to subjective analysis.

**Availability and implementation:**

The data used in the application are from the Human Genome Diversity Project (HGDP) and is publicly available. Algorithms were implemented using the R software R Core Team (*R: A Language and Environment for Statistical Computing*. Vienna (Austria): R Foundation for Statistical Computing, 2020.) in the R package blockcpd, found at https://github.com/Lucas-Prates/blockcpd.

## 1 Introduction

In diploid organisms, such as humans, each individual’s genome is organized into pairs of chromosomes, each half inherited from each parent. When an individual is an offspring of biologically related parents, both chromosomes of the same pair can share identical segments, creating long stretches of consecutive homozygosity, known as runs of homozygosity (ROH).

In the last decades, studies on the identification of ROH carried out in human populations have revealed the presence of ROH even in cosmopolitan non-inbred populations, disclosing an increment of inbreeding levels and the consequent reduction of genetic diversity of populations, which is proportional to the walking distance from Africa, as expected by the out-of-Africa model of human colonization (Kirin et al. 2010, Leutenegger et al. 2011, Pemberton et al. 2012, Ceballos et al. 2018, Lemes et al. 2018).

The distribution of ROH along the chromosomes is very uneven, resulting in some genomic regions having significant absence (coldspots) or excess of ROH (ROH islands) (Ceballos et al. 2018). The mechanisms for the emergence of these regions are still under discussion. For example, there is evidence that ROH islands could represent regions that harbor genes target of positive selection since low-recombination regions commonly are locations of selective sweeps, in which a new beneficial mutation increases in frequency and becomes fixed, causing the overall reduction in genetic diversity of the region (Pemberton et al. 2012, Ceballos et al. 2018).

To detect ROH and ROH islands, the genetic material of individuals from a given population is genotyped, and a set of single nucleotide polymorphisms (SNPs) is obtained. Each SNP entry is codified to 1 if that SNP belongs to an ROH for that individual and to 0 otherwise, where a marker is defined as belonging to an ROH for an individual if it is surrounded by a region with high frequency of homozygous SNPs. Finally, ROH islands are regions in which ROH are most frequent in that population. That is, the positions in the array with high frequency of individual ROH passing through them. Therefore, we can think on the problem of ROH island detection in a population as the identification of regions with high frequencies of 1’s in the codified SNPs of individuals of that population. That is, this problem can be regarded as a change-point problem for the parameters of a multidimensional random vector with Bernoulli marginal distributions.

### 1.1 Change-point detection

Classically, change-point detection refers to the problem of determining the times at which sequential observed data undergoes an abrupt change. In that type of setting, a change-point may refer to changes in mean (Page 1954, Tsay 1988, Keshavarz et al. 2018), variance (Chen and Gupta 1997, Hawkins and Zamba 2005), regression slope (Chow 1960, Qu and Perron 2007), general distributions forms (Matteson and James 2014), or other types of change (Castro et al. 2018, Leonardi et al. 2021). Many of these methods have been applied to a wide range of problems such as stream anomaly detection in industry (Li et al. 2018), monitoring of sleep stages using EEG/EMG (Agudelo-España et al. 2020), identification of cyberattacks on networks (Tartakovsky et al. 2006), between many other interesting applications. For more references about change-point methods for time series and other applications, we refer the reader to the book Chen and Gupta (2012) or the reviews Truong et al. (2020) and Lee (2010).

As Niu et al. (2016) points out, from a methodological point of view, there is a variety of ways to formulate the change-point detection problem: online versus offline, single change-point versus multiple change-points, parametric versus nonparametric, Bayesian versus non-Bayesian, and many other approaches when we dive into specific estimation procedures. But in most of the classical formulations for change-point detection, the estimation problem is considered under the hypothesis that the number of observations along the dimension of interest grows. Early results of Hinkley (1970) show that, even for more straightforward problems, these methods are usually consistent for the change-points fractions, but not the change-points properly. For offline issues, a possible interpretation is that we have a fixed number of series (usually one) over a unit interval. What grows with the number of observations, in this case, is the resolution over this interval.

As technology advances and other types of data arise from different application areas, new types of change-point detection problems arise as well. As we will see, ROH islands detection for a population is an example of this. For this problem, the dimension of interest is discrete and finite, and what grows is the number of independent observations of the random vector. Moreover, since the indices correspond to specific positions such as biological markers, it is essential to recover the exact location of the change-points. Therefore, it is more appropriate to have theoretical consistency to the true change-points as the number of independent observations of the joint multidimensional distribution grows.

### 1.2 Our contribution and related works

This work considers several aligned, independent samples of a multidimensional distribution, and we assume the distribution has a block structure with different parameters on each block. We focus on detecting such changes in the parameters, that is, the boundaries of the blocks when the number of observations grows, without assuming the number of blocks is known *a priori*. We aim to jointly estimate the number and the position of the change-points in the sample.

We propose a penalized maximum likelihood approach to detect the set of change-points in the distribution. General distribution-free conditions for the consistency of the exact estimator using dynamic programming and the greedy efficient estimator using binary segmentation are presented. Indeed, proving the consistency of the binary segmentation algorithm is a complex problem in change-point theory, and we prove its consistency using techniques different from previous works by Rice and Zhang (2022), Fryzlewicz (2014) and Venkatraman (1992). To show that our framework is reasonable, we verify the conditions leading to the consistency of the estimators for standard distributions such as categorical random variables and Gaussian random variables. Still, other families of distributions can satisfy these conditions, widening the possibility of applying our method to many different applied problems.

Another interesting contribution to the theory is the introduction of the First Repeated Value (FRV) methodology to select the penalizing constant. The selection of the penalizing constant for this type of estimator is a complex problem in practice and can drastically impact its performance. Albeit reference methodologies as provided by Lavielle (2005) and Haynes et al. (2017) exist, they have drawbacks such as relying on visual inspection. Our methodology is fully automatic and still preserves the consistency of the algorithms.

We discuss the identification of the ROH islands in the African and European populations based on the analysis of SNP data from the Human Genome Diversity Project (HGDP) (Li et al. 2008). We compare the ROH islands detected by our method with those detected by the procedure proposed in McQuillan et al. (2008); Kirin et al. (2010), using the PLINK software (Purcell et al. 2007), a well-established command-line software designed to solve many medical and population genetics problems.

## 2 System and methods

Let *m* be a positive integer and consider a random vector $X=(X_{1},\ldots ,X_{m})$ taking values in $A^{m}$, where *A* is any state space. Let Θ be a parametric space and consider a family $F=\{f_{\theta }|\theta \in \Theta \}$ of probability densities or probability mass functions over $A^{\ell }$, for each ℓ∈N, indexed by θ∈Θ. We assume each variable $X_{i}$, i=1,…,m is distributed according to some $f_{\theta }\in F$. Given two integers *r* and *s*, with r≤s, we use the notation r:s for the set {r,r+1…,s}. Denote by C the class of all ordered sets $C=\{c_{0},c_{1},\ldots ,c_{k}\}\subseteq 0:m$ such that $c_{0}=0$ and $c_{k}=m$. We say that C∈C is a change-point set for the random vector X if the variables in the subvector $\left(X_{c_{j-1}+1},\ldots ,X_{c_{j}}\right)$ are identically distributed with distribution $f_{\theta_{j}}\in F$ and $\theta_{j}\neq \theta_{j+1}$ for all j=1,…,k−1. Observe that the change-point vector is unique, and from now on it will be denoted by $C^{*}$. Given any set C∈C, we denote by $k_{C}$ the number of positive elements in *C*; that is, if $C=\{c_{0},c_{1},\ldots ,c_{k}\}$ then $k_{C}=k$.

Assuming the blocks in the random vector X are independent, we can write the probability of observing $x=(x_{1},\ldots ,x_{m})\in A^{m}$ in the model with parameters (C,θ), $\theta =\{\theta_{j}\in \Theta :j\in 1:k_{C}\}$ by: where $f_{\theta_{j}}(x_{(c_{j-1}+1):c_{j}})$ represents the distribution of a random vector over $A^{c_{j}-c_{j-1}}$ with parameter $\theta_{j}$. The independence assumption over the different blocks is not a necessary condition for the method but the generalization to a nonindependent setting is out of the scope of this work.


(1) $$P_{\left(C,\theta \right)}(x)=\prod_{j=1}^{k_{C}}f_{\theta_{j}}(x_{(c_{j-1}+1):c_{j}}),$$

Consider a sample of *n* i.i.d. random vectors $x^{n}={\left\{x^{\left(i\right)}\right\}}_{i=1}^{n}$ distributed as X, with change-point set $C^{*}=(c_{0}^{*},\ldots ,c_{k^{*}}^{*})$ and parameters $\theta^{*}=(\theta_{1}^{*},\ldots ,\theta_{k^{*}}^{*})$. Our main goal is to estimate the change-point set $C^{*}$ and the parameters $\theta^{*}$.

For any integer interval I⊂1:m assume, we can compute the maximum likelihood estimator based on the subsample ${\left\{x_{j}^{\left(i\right)}\right\}}_{i\in 1:n,j\in I}$. Write the maximum likelihood function for the set *C* of candidate change-points as: where ${\hat{\theta }}_{j}$ denotes the maximum likelihood estimator computed on the sample ${\left\{x_{c}^{\left(i\right)}\right\}}_{i\in 1:n,c\in I_{j}}$ with $I_{j}=(c_{j-1}+1):c_{j}$. From (2) the log-likelihood function is given by:


(2) $$L(C;x^{n})=\prod_{i=1}^{n}P_{\left(C,\hat{\theta }\right)}(x^{\left(i\right)})=\prod_{i=1}^{n}\prod_{j=1}^{k_{C}}f_{{\hat{\theta }}_{j}}\left(x_{(c_{j-1}+1):c_{j}}^{\left(i\right)}\right)$$

(3) $$l(C;x^{n})=\sum_{i=1}^{n}\sum_{j=1}^{\left|C\right|}\;\text{log}\;f_{{\hat{\theta }}_{j}}(x_{(c_{j-1}+1):c_{j}}^{\left(i\right)}).$$

Let R:C→R denote some regularization function and J:N→R an increasing function on the sample size *n*. We introduce the penalized likelihood estimator based on the functions *R* and *J* in the following definition.

Definition 1. *Given a sample* $x^{n}$*and a constant* λ>0*, the Penalized Likelihood (PL) function for the set of change-points C is defined as*:

*The PL estimator for the change-point set is then defined as*:

As we will show later in Theorems 1 and 2, in order to obtain the consistency of the change-point estimator defined by (5), we need the functions *R* and *J* to satisfy some properties. This will be made precise in the statements of these theorems.

(4) $$\text{PL}(C;x^{n})=-l(C;x^{n})+\lambda R(C)J(n).$$

(5) $$\hat{C}(x^{n})=\underset{C\in C}{\text{argmin}}\text{PL}(C;x^{n}).$$

## 3 Algorithms

In order to efficiently estimate the change-point set, we suppose in this section that the regularization function *R* is additive. That means that there exists a function $\rho :{\left\{1,\ldots ,m\right\}}^{2}\to R$ such that for $C=\{c_{0},\ldots ,c_{k}\}$ we have:


(6) $$R(C)=\sum_{j=1}^{k_{C}}\rho (c_{j-1}+1,c_{j}).$$

### 3.1 Dynamic programming segmentation algorithm

As presented in Jackson et al. (2005), dynamic programming can be used to calculate exactly the PL estimator. Under an additive regularization, the function we want to minimize can be written as: where


$$\begin{array}{c} -l(C;x^{n})+\lambda J(n)R(C)=\sum_{j=1}^{k_{C}}Q((c_{j-1}+1):c_{j}), \end{array}$$

$$Q((c_{j-1}+1):c_{j})=-\text{log}\;f_{{\hat{\theta }}_{j}}(x_{(c_{j-1}+1):c_{j}}^{\left(i\right)})+\lambda J(n)\rho (c_{j-1}+1,c_{j}).$$

The equation shows that we can completely decouple the loss from different blocks. Let $C_{i}$ be the set of all ordered change-point sets in 1:i. Define as the optimal value for the segmentation up to variable *i*. The estimator $\hat{C}(x^{n})$ given by (5) is obtained by computing F(m). But notice that which establishes a recursion equation for the values of F(i), *i* varying from 1 to *m*. The value of F(1) can be computed trivially, and then we use the recursion to compute the values until we reach F(m).


$$F(i)=\underset{C\;\in \;C_{i}}{\min}\{\sum_{j=1}^{k}Q((c_{j-1}+1):c_{j})\}$$

$$F(i)=\underset{c\in (k-1):(i-1)}{\min}\{F(c)+Q((c+1):i)\},$$

Albeit *m* is fixed and only *n* grows, the number of variables *m* can be very large in some applications, so it is useful to express the complexity in terms of both. The dynamic programming segmentation algorithm runs on a time complexity of $O(m^{2})$. However, we are assuming that *Q* have been previously computed for all intervals in 1:m. To compute *Q*, we need to compute the maximum likelihood estimators for each block. For most models, this can be done efficiently by computing the sufficient statistics, and then compute the entries of *Q*. In the case where no fixed dimension sufficient statistics exist, we need to reprocess the data every time, so the complexity to compute *Q* is $O(nm^{3})$. Hence, the final time complexity of the algorithm is $O(T(n,m)+m^{2})$. In the worst case the algorithm is $O(nm^{3})$, and can be very slow for big values of *m*.

Depending on the function ρ chosen, the PELT algorithm of Killick et al. (2012) might be applicable. It consists of an adaptation of the dynamic programming algorithm discussed here. In some scenarios, such as when the number of change-points is proportional to the number of variables, it runs in O(m). The final complexity would be O(nm+m) when suitable sufficient statistics exist.

### 3.2 Hierarchical segmentation algorithm

For efficient computation of $\hat{C}(x^{n})$ we can use an approximation to the optimum in (5), known as hierarchical segmentation or binary segmentation, first proposed in Scott and Knott (1974). Given an integer interval I=r:s, write $x_{I}^{n}$ for the data with columns restricted to *I* and assume the penalizing function *R* is additive. Define the penalized loss of *I* as: with the appropriate renumbering of the columns in $x_{I}^{n}$. That is, the penalized loss corresponding to the interval *I* is the penalized loss defined in (4) when we only consider the data $x_{I}^{n}$ and perform no splits. We use the convention PL(∅)=0. For c∈I=r:s, define:


$$\text{PL}(I)=\text{PL}(\{0,s-r+1\};x_{I}^{n})=-l(\{0,s-r+1\};x_{I}^{n})+\lambda \rho (r,s)J(n),$$

(7) $$h_{I}(c)=\text{PL}(r:c)+\text{PL}((c+1):s).$$

Observe that when c=s, by convention, we have (s+1):s=∅ so that $h_{I}(s)=\text{PL}(I)$.

The hierarchical segmentation algorithm works recursively as follows. It begins with the set ${\hat{C}}_{hs}(x^{n})=\{0,m\}$ corresponding to the single interval I=1:m. In each iteration and for each interval *I* determined by ${\hat{C}}_{hs}(x^{n})$, the algorithm computes $\hat{c}=\underset{c\in I}{\text{argmin}}\;h_{I}(c)$ and adds it to ${\hat{C}}_{hs}(x^{n})$. Observe that if in one iteration $\hat{c}=s$, as $s\in {\hat{C}}_{hs}(x^{n})$, no changes are made on ${\hat{C}}_{hs}(x^{n})$. The process continues until no more points can be added to ${\hat{C}}_{hs}(x^{n})$.

To evaluate PL at each interval, we either store all possible values in the same fashion as for the dynamic programming algorithm or we evaluate them on the run. Since storing would make the algorithm $O(m^{2})$ in any scenario, it is more interesting to precompute the sufficient statistics and evaluate the loss on the intervals as they appear.

In the worst-case scenario, the algorithm has time complexity of order $O(T(n,m)+m^{2})$. However, the algorithm has asymptotic complexity of order $O(T(n,m)+mk_{C^{*}}))$, as proved in the Supplementary Material.

### 3.3 Consistency of the algorithms

In this section, we state the theoretical results that guarantee the consistency of the estimator (5) computed exactly by the dynamic programming algorithm or approximated by the hierarchical segmentation algorithm. For each method, we state a different set of assumptions that must be satisfied by the family of probability distributions considered in the model.

Assumption 1. *Suppose there exists a function* $l^{*}:C\to R$*such that*

*(PL1) For any* C∈C*we have that* $\frac{1}{n}l(C;x^{n})\;\to \;l^{*}(C)$*almost surely as* n→∞*, where l is the log-likelihood function defined in (*3*). Moreover, assume there exists* α>0*such that*

*(PL2)There exists a sequence* ${\left\{v(n)\right\}}_{n\in N}$*satisfying* v(n)→∞*and* v(n)/n→0*when* n→∞*, and such that for any* $C⊇C^{*}$we have that $\left|l(C;x^{n})-l(C^{*};x^{n})|<v(n\right)$*eventually almost surely as* n→∞.

Observe that (PL2) implies that $l^{*}(C)=l^{*}(C^{*})$ for all $C⊇C^{*}$.

We now state the consistency result of the PL estimator given in (5).

Theorem 1. *Suppose that the family* F*of probability distributions satisfyAssumption 1. Let R be a penalizing function such that*$R(C)>R(C^{\prime })$*whenever* $C⊃C^{\prime }$*and let* J(n)*be such that* J(n)/v(n)→∞*and* J(n)/n→0*when* n→∞*. Then the estimator of the change-point set given by (5) satisfies* $\hat{C}(x^{n})=C^{*}$eventually almost surely as n→∞.

infC⊇C*l*(C)≤l*(C*)−α<l*(C*).

Notice that the regularization function *R* does not need to be additive to guarantee the consistency of the PL estimator. However, this is a desirable property to efficiently compute the estimator by using the dynamic programming segmentation algorithm.

In order to prove that the estimator given by the hierarchical segmentation algorithm is also consistent, we need a slightly different set of hypotheses considering the local nature of this algorithm. Denote by I the set of all intervals I⊂1:m. Given I∈I, denote by ${\hat{\theta }}_{I}$ the maximum likelihood estimator of the parameter θ on the interval *I* and as before, let $x_{I}^{n}$ be the data restricted to the interval *I*. Define the maximum log-likelihood function for the interval *I* as:


$$l(I;x_{I}^{n})=\sum_{i=1}^{n}\;\text{log}\;f_{{\hat{\theta }}_{I}}(x_{I}^{\left(i\right)}).$$

Let $h_{I}$ be the loss function for the interval *I* as defined in (7).

Assumption 2. *There exists a function* $l^{*}:I\to R$*such that*

*(H1)For any integer interval* I∈I*we have that* $\frac{1}{n}l(I;x_{I}^{n})\;\to \;l^{*}(I)$*almost surely as* n→∞*. If* I=r:s*, defining* $h_{I}^{*}:I\to R$*as*: *we have that* minc∈I∖{s}∩C* hI*(c)<minc∈I∖{s}∩C* hI*(c).

*(H2)There exists a sequence* ${\left\{v(n)\right\}}_{n\in N}$*satisfying* v(n)→∞*and* v(n)/n→0*when* n→∞*, and such that, for any integer interval* I=r:s*satisfying* $I\setminus \{s\}\cap C^{*}=\emptyset$*we haveeventually almost surely as* n→∞.

(8) $$h_{I}^{*}(u)=-l^{*}(r:u)-l^{*}((u+1):s),\;u\in I,$$

$$\underset{u\in I}{\max}\;|l(I;x_{I}^{n})-l(r:u;x_{I}^{n})-l((u+1):s;x_{I}^{n})|<v(n)$$

We can now state the consistency of the change-point set estimator given by the hierarchical segmentation algorithm.

Theorem 2. *For any* λ>0*, let* ${\hat{C}}_{hs}(x^{n})$*be the estimator computed by the hierarchical segmentation algorithm. Suppose that the family* F*of probability distributions satisfyAssumption 2. Suppose that R satisfies (6) for some function*ρ:I→R*and that* $\rho (I)<\rho (I_{1})+\rho (I_{2})$*whenever* $I=I_{1}\cup I_{2}$*, with* $I_{1},I_{2}\neq \emptyset$*. Finally, assume that the function* J(n)*satisfies* J(n)/v(n)→∞*and* J(n)/n→0*when* n→∞*. Then*, ${\hat{C}}_{hs}(x^{n})=C^{*}$eventually almost surely as n→∞.

The proof of Theorems 1 and 2 are included in the Supplementary Material to this article, available online. There, we also show that the family of Bernoulli and Gaussian random variables satisfy Assumptions 1 and 2. Hence, both the dynamic programming and hierarchical segmentation algorithms provide consistent estimators of the change-point parameters $\left(C^{*},\theta^{*}\right)$ for these families of distributions.

### 3.4 FRV methodology for penalizing constant selection

Albeit the algorithms are consistent for any value of the penalization constant λ, their performance, in practice, can drastically improve if we choose good values of the constant. To this end, a reference methodology was proposed by Lavielle (2005) and efficiently computed in the CROPS algorithm by Haynes et al. (2017). The idea consists of plotting the cost function, the negative log-likelihood, versus the number of change-points and selecting the value at which the curve starts to become flat, a technique similar to the elbow plot used in cluster analysis. This approach is widely used in practice; however, it has some drawbacks. For instance, it requires visual inspection, hence human intervention, making it nonautomatic. Because of this, one cannot study the consistency of the whole procedure. Moreover, the correct choice of the number of change-points is difficult due to the cost function’s lack of a reference scale.

To address these issues, we propose a similar idea to that of the elbow plot, but on different variables. Instead of plotting the cost function versus the number of change-points, we see how the proportion of change-points varies against the values of the penalization constant. Chosen γ>0 and $\lambda_{\text{max}}>0$, the idea is to evaluate the detected number of change-points for each constant value in the grid {0,γ,2γ…,bγ}, where $b=\left\lfloor \frac{\lambda_{\text{max}}}{\gamma }\right\rfloor$.

In order to simplify the notation, let $\hat{l}(k)$ be the log-likelihood of the best split with *k* change-points evaluated at the MLE and R(k) be the regularization of the best change-point set. Fixed λ, consider the function $PL(k)=-\hat{l}(k)+\lambda J(n)R(k)$ as the Penalized Likelihood for the best model with *k* change-points. If, for every λ, the function is strictly convex, then the model estimates $\hat{k}=k$ if, and only if, both equations below hold:


$$\begin{array}{c} -\hat{l}(k)+\lambda J(n)R(k)\leq -\hat{l}(k+1)+\lambda J(n)R(k+1)\\ -\hat{l}(k)+\lambda J(n)R(k)<-\hat{l}(k-1)+\lambda J(n)R(k-1). \end{array}$$

For each *k*, define the interval making the convention that this interval is empty if the left extreme is greater than the right one. The method selects $\hat{k}=k$ if, and only if, we have λ∈A(k).


$$A(k)=\left[\frac{\hat{l}(k+1)-\hat{l}(k)}{J(n)(R(k+1)-R(k))},\frac{\hat{l}(k)-\hat{l}(k-1)}{J(n)(R(k)-R(k-1))}\right),$$

Under the assumptions of Theorem 1 or Theorem 2, we have $|A(k)|\in O\left(\frac{v(n)}{J(n)}\right)\overset{a.s.}{\to }0$ if $k>k^{*}$, $A(k^{*})\overset{a.s.}{\to }(0,+\infty 0$ and $|A(k^{*})|\in O\left(\frac{n}{J(n)}\right)\overset{a.s.}{\to }+\infty$, where |A(k)| denote the length of the interval A(k). The behavior for A(k) for $k<k^{*}$ is unimportant for the procedure.

Choose the step size γ=γ(n) such that $\frac{v(n)}{J(n)}\ll \gamma (n)$ and γ(n)→0 as n→∞. Taking a sample size that is large enough, if at the grid point iγ the algorithm outputs $k>k^{*}$, then at the point (i+1)γ it will output $k^{\prime }$ such that blue$k>k^{\prime }\geq k^{*}$. This is due to the fact that the step size is greater than the interval length |A(k)|. If the algorithm outputs $k^{*}$, then at the next point it will output $k^{*}$ again, now due to γ being smaller than $\left|A(k^{*})\right|$. Therefore, the key idea is to fit the models until we get a repeated value for the estimated number of change-points. If no repeated values occur in the given interval, we can just halve γ and evaluate in the new grid until a repeated value appears.

The First Repeated Value (FRV) procedure outputs the penalization value and model such that the first repeated value of the number of change-points is detected. The pseudocode is given below. Assume we have a procedure fitModel that fits the model for a given penalization constant and getNCP that extracts the number of change-points.

Algorithm 1 First Repeated Value procedure

 **procedure** FRV(X, γ, $\lambda_{\text{max}}$, m)

  **while** True **do**

   lastNCP←1  ▹ at λ=0 the proportion is always 1

   $b\leftarrow \left\lfloor \frac{\lambda_{\text{max}}}{\gamma }\right\rfloor$

   **for**i∈{1,…,b}**do**

    model←fitModel(X,iγ)

    currNCP←getNCP(model)/m

    **if**lastNCP=currNCP**then**

     return (model,currNCP)  ▹ output the model

    **end if**

    lastNCP←currNCP

   **end for**

   γ←γ/2   ▹ halves γ since no repetition was found

  **end while**

 **end procedure**

To exemplify how γ(n) can be chosen, take J(n)=log(n) and consider the families of Normal distributions with unknown mean and variance. In the Appendix and in the Supplementary Material, we show that v(n)=O(log(log(n)). Then we can take $\gamma (n)={\left(\text{log}(n)\right)}^{-1/2}$ and we have that $\frac{v(n)}{J(n)}\ll \gamma (n)$, with γ(n)→0 as n→∞.

We now state a theorem on the consistency of the FRV procedure. The proof is presented in the Supplementary Material.

Theorem 3. *Assume that the model satisfies the consistency conditions for eitherTheorem 1 or Theorem 2. Let*$\lambda_{\text{max}}>0$*be constant and assume* $\frac{v(n)}{J(n)}\ll \gamma (n)$*and assume that, for every constant* λ*, the Penalized Likelihood* $PL(k)=-\hat{l}(k)+\lambda J(n)R(k)$*is strictly convex. Let* ${\hat{C}}_{\mathrm{FRV}}$*be the change-point set estimated by the FRV procedure. Then, we have* ${\hat{C}}_{\mathrm{FRV}}=C^{*}$eventually almost surely as n→∞.

This approach has the stated properties; first, the proportion of change-points always varies from 1 to 0, so that it is scale free from the statistical family, sample size, and number of change-points; second, it allows us increasingly refine the penalization grid; third, it allows for an automatic constant selection method that is consistent.

## 4 Implementation

The exact dynamic programming algorithm and the hierarchical segmentation algorithm described in Section 3 were implemented using the R software R Core Team (2020) in the R package blockcpd. The performance of both algorithms was compared to the classical CUSUM method for sequence segmentation on model-based simulated data. As the hierarchical algorithm showed similar performance to the exact approach and is computationally more efficient, it was applied to a real SNPs dataset to detect ROH islands. The results were confronted against the ROH islands detected by PLINK and by Pemberton et al. (2012) (results available in the Supplementary Material).

### 4.1 Simulations

In the model-based simulations, we consider two families of distributions: the Bernoulli distribution and the Gaussian distribution, with both mean and variance unknown (results available in the Supplementary Material). We fixed the number of variables *m* and varied the number of change-points in the model.

The simulated datasets were generated as follows. For each probability distribution (Bernoulli and Normal), we varied the number of samples *n* from 50 to 500 in steps of 50. For each sample size, we simulated 1000 datasets with *n* samples. We fixed the number of variables as m=200 and the number of change-points $|C^{*}|=k^{*}$ to take values in {10,50}. For each $k^{*}$, the change-points were sampled without replacement from a uniform distribution in *[*1, 199*]* and where maintained fixed for all datasets and all sample sizes. Bernoulli parameters were sampled independently from a uniform distribution. For the Gaussian distribution, means were sampled independently from a $N(0,\sqrt{5})$, and variances from an Exp(1). These parameters also remained fixed for all datasets and all sample sizes.

For each dataset, we computed the change-points with both the dynamic programming segmentation algorithm and the hierarchical segmentation algorithm, as described in Section 3. In order to select the penalization constant, we applied the FRV methodology for the interval *[*0, 10*]* with steps $\gamma =\frac{1}{\sqrt{\;\text{log}(n)}}$.

In order to compare our methods with the traditional approach CUSUM, we applied the changepoint package from Killick and Eckley (2014) on the series consisting of the average of each variable (column). We used a mean-variance change-point detection algorithm assuming normality, considering the PELT search algorithm and the BIC as a regularization.

To evaluate the convergence of the change-point set estimated by the algorithms, we considered the Jaccard Index, defined by $J(C_{1},C_{2})=\frac{\left|C_{1}\cap C_{2}\right|}{\left|C_{1}\cup C_{2}\right|}$. We also took into account the convergence of the estimated number of change-points to $k^{*}$. It allow us to see when the model is heavily underestimating or overestimating the number of change-points.

**Figure 1.**
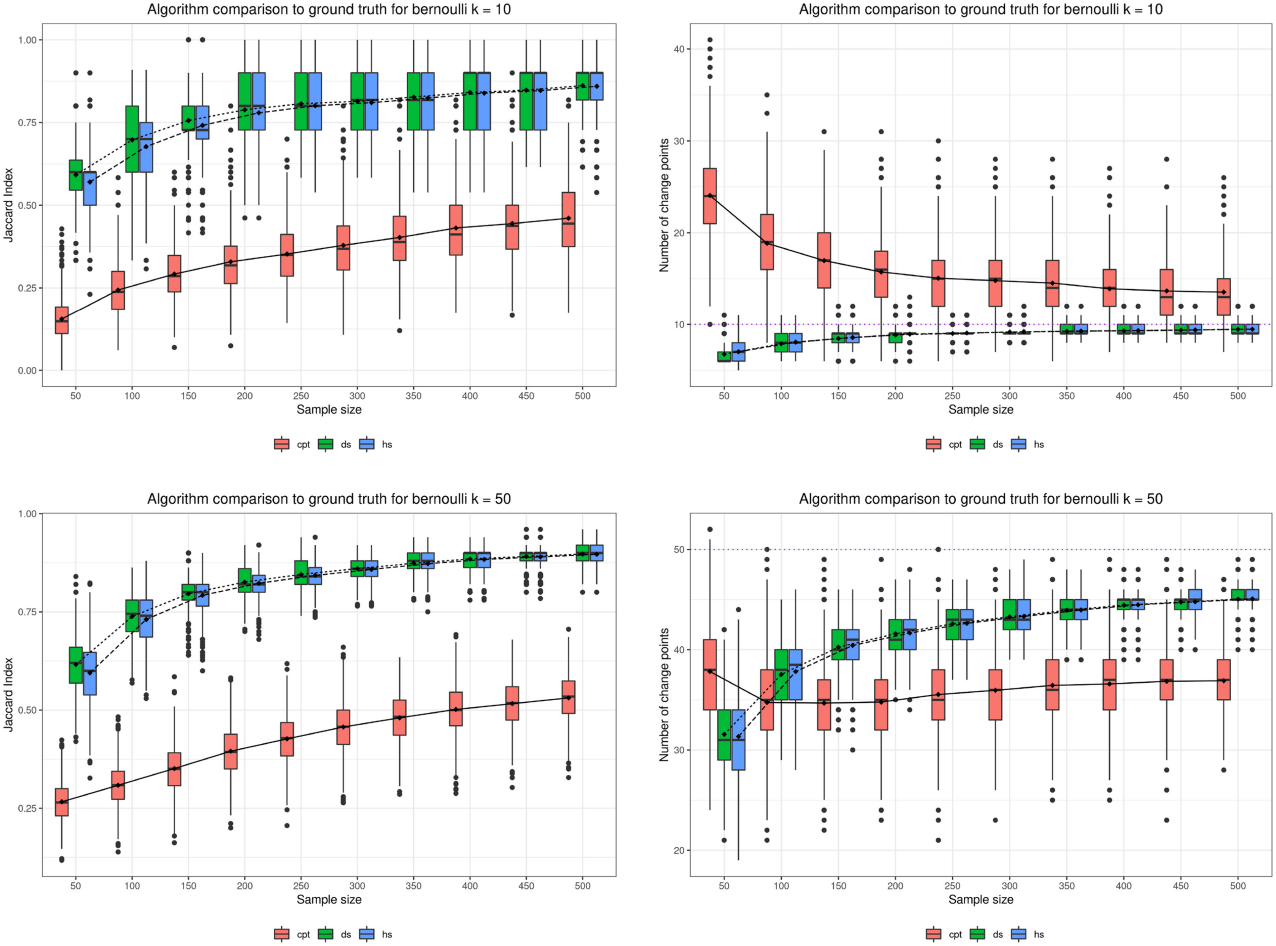
Comparison of estimated change-point sets between CUSUM (cpt), dynamic programming segmentation (ds), and hierarchical segmentation (hs) algorithms for the Bernoulli family using the Jaccard Index (left) and the number of change-points (right). The true number of change-points *k* is indicated by the dotted horizontal line

Figure 1 shows the comparison of the performance of the algorithms as the sample sizes grow. The boxplots median is approximating the true number of change-points and 1 for the Jaccard Index. Moreover, they are becoming increasingly narrower and closer to 1, which indicates total similarity between the change-point sets. The average lines are also shown to help us envisage the behavior of the algorithms as sample size increases.

For the Bernoulli and Normal simulation scenarios, the approximate algorithm has very similar performance to the exact dynamic programming algorithm. Indeed, their boxplots are almost indistinguishable. For small sample size, as expected, the hierarchical algorithm exhibits greater variance.

In the Bernoulli scenario, the CUSUM method performs much worse than our algorithms, as indicated by Fig. 1. This is expected since our statistical model is correct for the data in this case, and the CUSUM method relies on the normal approximation to detect changes in mean and variance. For the Normal scenario, when $k^{*}=10$, the CUSUM method is very competitive and shows smaller variance than our algorithms. For $k^{*}=50$, our algorithms outperform again the CUSUM method.

It is also important to mention that the FRV methodology plays a key role in selecting the constant in the simulation. Indeed, it would be a burdensome task to perform visual inspection for each dataset. The models are being fit in an increasing grid of penalization constants. As shown by the simulation results, the FRV is automatically selecting models with increasingly better performance as the sample size grows.

### 4.2 ROH islands on African and European populations

As described in the introduction, we propose to frame the problem of ROH islands detection as a change-point detection problem assuming a Bernoulli marginal distribution for each codified SNP. Observe that we do not need to assume independence between different SNPs in order to have consistent estimators of the change-points, we only assume consecutive parameters on the blocks of the distribution to be different. In particular, the ROH islands can be determined as those blocks with a high value of the estimated parameter.

We can use domain knowledge to construct a proper regularization function. The first consideration is that the distance between SNPs is not uniform. That is, the distance between the *i*-th and *j*-th SNP is not |i−j|, but rather |B(i)−B(j)|, where *B* is a function that maps each SNP to its physical location on the chromosome. The physical location of a SNP is measured as the number of base pairs before that particular SNP. The second observation is that very small blocks are usually not interesting for the analyst. It is usual to set a minimum block size in which SNPs are grouped.

Considering these observations, we define the regularization function ρ for the block r:s as:


$$\rho (r,s)=\{\begin{array}{cc} +\infty & \;\;\text{if}\;\frac{\left|B(s)-B(r)\right|}{\beta }\leq T\\ \frac{1}{|B(s)-B(r)|/\beta } & \;\;\text{otherwise}. \end{array}$$

In the expression above, *T* denotes a threshold for the minimal physical distance allowed for an ROH island, and $\beta =10^{6}$ is a scaling factor to work on mega bases unit. The regularization function R(C) in (4) is then defined as the sum of the function ρ over the different blocks in *C*, as in (6).

The SNPs data we analysed was obtained from the Human Genome Diversity Project (HGDP), and consists of approximately 600 000 SNP markers from Illumina HuHap 650k platform (Li et al. 2008). We considered individuals from African and European populations. On this dataset, each row represents an individual from the population, and each column corresponds to each SNP.

We estimated the change-point set and parameters on each block for each population using a value of λ that was selected using the FRV in the interval *[*0, 10*]* with steps of $\gamma =\frac{1}{\sqrt{\;\text{log}(n)}}$. The threshold size *T* was set to be 1% of the chromosome size, and the penalization for the sample size set to $J(n)=\sqrt{n}$.

For each population, we also performed ROH identification for each individual with the criteria described by McQuillan et al. (2008) and Kirin et al. (2010), using the software PLINK v1.9 (Purcell et al. 2007).

**Figure 2.**
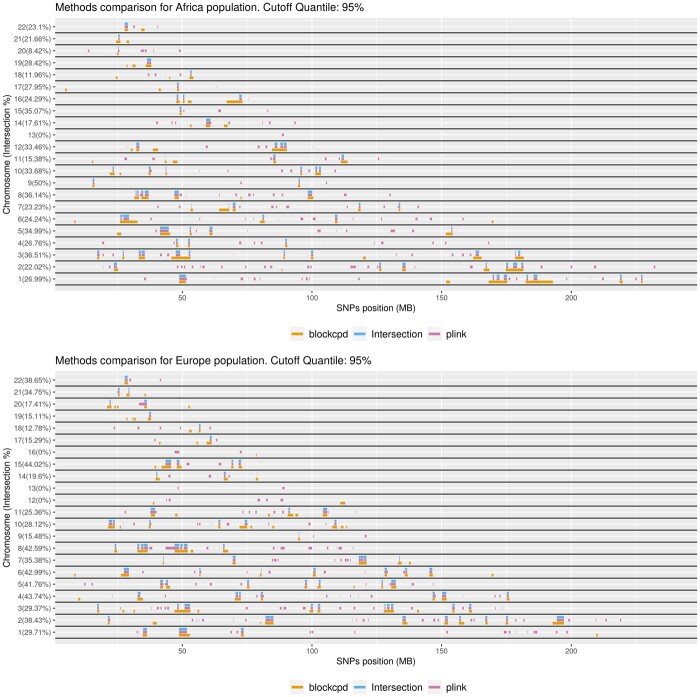
Comparison of the detected ROH islands across the genome by our hierarchical algorithm (blockcpd) and the PLINK method. Both ROH islands detected by PLINK and our method are shown, together with their intersection. The intersection percentage is displayed on the vertical axis

Figure 2 shows the ROH islands, for African and European populations, detected across the genome by considering a quantile cutoff of 95%. For PLINK, the cutoff is based on the frequency distribution that SNPs appear in ROH. For blockcpd, the cutoff is based on the probability parameter of the blocks. We see an overlap between the estimates for almost all chromosomes. The intersection percentage is shown on the vertical axis. We observe that PLINK tends to subdivide regions, while our method provided more contiguous estimates, as was the goal when we introduced the regularization function and the threshold parameter *T*.

A comparison of the ROH islands estimates for a quantile cutoff of 99% is provided in the Supplementary Material, where we also included the analysis of full genome sequences from the HGDP database and the comparison of our method with the method by Pemberton et al. (2012).

## 5 Discussion

In this paper, we proposed a new change-point detection method based on penalized maximum likelihood and proved its consistency, for two different algorithms. This new approach is motivated by the problem of identifying homozygosity islands on the genome of individuals in a population. Our method directly tackles the issue of determining the homozygosity islands at the population level without analysing single individuals and then combining the results, as is made nowadays in state-of-the-art approaches. Applying this method to real data of two populations from the Human Genome Diversity Project (HGDP) showed the potentiality of these algorithms to highlight highly homozygous regions in the genome. The nonhomogeneity of the regularization function *R* can provide flexibility to incorporate more domain knowledge of the application area.

We also propose the FRV methodology to automatically and efficiently select the penalization constant λ. We proved that the methodology preserves the consistency property even if fitting the model in an increasing grid of penalization constants. The simulation study shows that the methodology chooses models with overall good performance.

From the theoretical point of view there is much to explore in future research. A first step will be to check if the assumptions implying the consistency results hold for a wider class of distributions, such as the Exponential Family, finite-state Markov Chains and Multivariate Gaussian distributions within each block. A second interesting question is to prove or give a counter-example of whether Assumption 2 implies Assumption 1; that is, if the convergence of the greedy algorithm implies the convergence of the exact algorithm as well.

Conflict of interest: None declared.

## Supplementary Material

btad170_Supplementary_Data

## Data Availability

The data underlying this article are available in the Human Genome Diversity Project (HGDP) at https://www.science.org/doi/10.1126/science.aay5012. The datasets were derived from sources in the public domain: ftp://ngs.sanger.ac.uk/production/hgdp/hgdp_wgs.20190516/hgdp_wgs.20190516.full.chr[i].vcf.gz (i = 1 to 22).
