# Supplementary material for: Population-based change-point detection for the identification of homozygosity islands
Source: Bioinformatics. 2023 Apr 11;39(4):btad170. doi: 10.1093/bioinformatics/btad170 (PMC10112956; doi:10.1093/bioinformatics/btad170)
Supplement: btad170_Supplementary_Data [file btad170_supplementary_data.pdf]

Supplementary material to the article "Population-based change-point detection for the identification of homozygosity islands"

Lucas Prates, Renan B. Lemes, Tábita Hünemeier and Florencia Leonardi

March 30, 2023

1 Proof of main results in the article

In this section we present the proofs of Theorems 1, 2 and 3 stated in the article.

Proof of Theorem 1. First we will prove that $\widehat{C}(\mathbf{x}^n)$ will almost surely contain C^*. Let $C \in \mathcal{C}$ such that $C \not\supseteq C^*$. Then by (PL1) in Assumption 1 we have that eventually almost surely

$$\frac{1}{n}l(C;\mathbf{x}^n) - \frac{1}{n}l(C^*;\mathbf{x}^n) \;<\; -\frac{\alpha}{2}\,.$$

On the other hand, as $J(n) = o(n)$ and $R(C)$ is bounded we have that

$$\lambda(R(C) - R(C^*))\frac{J(n)}{n} \;\to\; 0$$

as $n \to \infty$. Therefore, eventually almost surely we have

$$\mathrm{PL}(C^*;\mathbf{x}^n) \;<\; \mathrm{PL}(C;\mathbf{x}^n)\,.$$

As the number of $C \not\supseteq C^*$ is finite we have that eventually almost surely $\widehat{C} \supseteq C^*$. Lets prove now that $\widehat{C} \not\supset C^*$. Assume $C \supset C^*$ so that $R(C) > R(C^*)$ and $l^*(C) = l^*(C^*)$. Then, by (PL2) we have that

$$\begin{aligned}\mathrm{PL}(C^*;\mathbf{x}^n) - \mathrm{PL}(C;\mathbf{x}^n) \;&\leq\; v(n) + \lambda(R(C^*) - R(C))J(n)\\ &<\; 0\end{aligned} \tag{1}$$

eventually almost surely as $n \to \infty$. As a result we obtain that $\widehat{C}(\mathbf{x}^n) = C^*$ eventually almost surely as $n \to \infty$. □

We now present the proof of the consistency of the hierarchical estimator.

Proof of Theorem 2. The proof begins by showing that, at any given possible scenario, the algorithm takes a correct choice almost surely. Then, an inductive argument will guarantee that the algorithm is consistent. For every integer interval $I = r:s$, the possible scenarios are:

(a) There are no change-points in $I \setminus \{s\}$;

(b) There are change-points in $I \setminus \{s\}$.

The correct decision for the algorithm in case (a) is to halt, not performing more recursive calls for that interval. For case (b), the correct decision is to choose any of the change-points available and perform recursive calls on the sub intervals. We show that the algorithm will take the correct decision almost surely for both cases.
For case (a), suppose I has no change-points inside and let $u \in r:(s-1)$. By (H2) in Assumption 2 we have that

$$\begin{aligned}h_I(s) - h_I(u) \;&=\; -\left[l(I;\mathbf{x}_I^n) - l(r:u;\mathbf{x}_I^n) - l((u+1):s;\mathbf{x}_I^n)\right] +\\ &\quad+ J(n)\lambda\left[\rho(r,s) - \rho(r,u) - \rho(u+1,s)\right]\\ &<\; v(n) + J(n)\lambda\left[\rho(r,s) - \rho(r,u) - \rho(u+1,s)\right]\\ &<\; 0\,,\end{aligned}$$

eventually almost surely as $n \to \infty$, because $\rho(r,s) - \rho(r,u) - \rho(u+1,s) < 0$. Hence, no splitting will be done eventually almost surely, and the algorithm will not perform more recursive calls inside I.
For case (b) we have to prove that the algorithm will almost surely choose true change-points to split the interval. The inequality (H1) on Assumption 2 implies that, for any u that is not a change-point, there exists a change-point c^* in $I \setminus \{s\}$ such that

$$h_I^*(c^*) \;<\; h_I^*(u)\,.$$

Then as $J(n) = o(n)$ we have that eventually almost surely as $n \to \infty$

$$\frac{1}{n}h(u) - \frac{1}{n}h(c^*) \;\geq\; \frac{1}{2}(h^*(u) - h^*(c^*)) \;>\; 0\,,$$

and therefore $\hat{c} \in C^*$, eventually almost surely as $n \to \infty$.

We finish the proof by using mathematical induction on the number of variables m. If $m = 1$, then there are no change-points on the model, and the algorithm will not even have comparisons to make. Hence, the estimated change-point set will be empty by construction and therefore equal to the true set of change-points. Assume now that the algorithm is consistent for every vector of dimension $\tilde{m} <= m-1$. We will prove that it will be consistent for vectors of dimension m. The first run of the algorithm is on the interval $1:m$. By case (a), if there are no change-points on this interval, the algorithm will almost surely not split the interval and will halt. Hence, the estimated set will almost surely be equal to the true set of change-points. On the other hand, if there are change-points on the interval $1:m$, the proof in case (b) shows us again that the algorithm eventually almost surely takes the correct choice and splits the interval at a change-point $c \in C^*$. After the split, recursive calls are made on $1:c$ and on $(c+1):m$. But the length of these vectors is at most $m-1$, and by the induction hypothesis, the algorithm will eventually almost surely retrieve all the change-points in $1:c$ and $(c+1):m$ exactly. By a union of those change-point sets, we have that the final estimated change-point set is equal to C^* eventually almost surely as $n \to \infty$. □

Proof of Theorem 3. From the observations made in Subsection 3.4, under the assumptions of Theorem 1 for the exact estimator, respectively Theorem 2 for the binary segmentation estimator, and for a fixed penalisation constant, if $k > k^*$ then we have that $|A(k)| = O\left(\frac{v(n)}{J(n)}\right)$, $|A(k^*)| \in O\left(\frac{n}{J(n)}\right)$ and $A(k^*) \xrightarrow{a.s.} (0,+\infty)$. Consider that the sample size n is large enough so that $|A(k)| < \gamma(n)$ and $|A(k^*) \cap [0, \lambda_{\max}]| > 3\gamma(n)$. The algorithm starts at the grid point $\gamma(n)$. Since $A(0) < \gamma(n)$, we must have that the number of change-points at $\gamma(n)$ is greater than 0. We can repeat the same argument for all $k > k^*$ until the first grid point $i\gamma(n)$ such that the algorithm returns k^*. At the point $(i+1)\gamma(n)$, since $A(k^*) > 3\gamma(n)$, we must have that the algorithm outputs k^* again. But then, since it repeated the last number of change-points estimated, it returns k^* and the model associated. □

2 Auxiliary results

Verifying directly (H1) on Assumption 2 is usually hard. We now state a lemma that provides easier to verify conditions that imply the desired assumption.

Lemma 1. *Given an integer interval I assume that*

(a) There exists a point $c \in I$ such that $h_I^*(c) < h_I^*(s)$.

(b) The function h_I^* restricted to the intervals $[c_{j-1}^*, c_j^*]$, $j = 1, \ldots, k^*$, is concave.

(c) If h_I^* is constant in $[c_{j-1}^*, c_j^*]$, then it is equals to $h_I^*(s)$ on this interval.

Then, the function h_I^* satisfies that

$$\min_{c \in I \setminus \{s\} \cap C^*} h_I^*(c) < \min_{c \notin I \setminus \{s\} \cap C^*} h_I^*(c) .$$

Proof. Let $h_I^*(u)$ be the minimum value of h_I^* on I. First, notice that by (a), $h_I^*(u) \le h_I^*(c) < h_I^*(s)$, so the minimum is strictly smaller than the value of the function at the end of the interval. We now show that u must be a change-point in C^*. Suppose that $u \notin C^*$, and let $I_j^* = [c_{j-1}^*, c_j^*]$ be the unique interval that contains u. Since h_I^* is concave in $[c_{j-1}^*, c_{j+1}^*]$, if the minimum is attained at an interior point, then h_I^* must be constant on I_j^*. However, by (c), this would imply that $h_I^*(u) = h_I^*(s)$, which is a contradiction. Then $u \in C^*$ and

$$\min_{c \in I \setminus \{s\} \cap C^*} h_I^*(c) < \min_{c \notin I \setminus \{s\} \cap C^*} h_I^*(c) .$$

□

The following lemma shows that under general conditions on the function h_I^* verified in practice by many models, the conditions of Lemma 1 hold.

Lemma 2. *Let Θ be a convex subset of $\mathbb{R}^d$ for some $d \in \mathbb{N}$. For any interval $I \in \mathcal{I}$ define the parameter $\theta_I = \sum_{k=1}^{k^*} \frac{|I \cap I_k^*|}{|I|} \theta_r^*$. Assume the function h_I^* defined by (8) for $I = r : s$, is of the form $h_I^*(u) = (u - r + 1)\psi(\theta_{r:u}) + (s - u)\psi(\theta_{(u+1):s}) + \alpha$, with ψ a strictly convex function having second order derivatives in the interior of Θ and α a constant not depending on u. Then h_I^* satisfies the conditions (a)-(c) in Lemma 1.*

Before presenting the proof of Lemma 2 we state and prove the following basic lemma.

Lemma 3. *Let $f, g : \mathbb{R} \to \mathbb{R}$ be twice differentiable convex functions. If there exists a constant α such that*

$$f(x) + g(x) = \alpha ,$$

then f and g must be linear functions.

Proof. Differentiating both sides twice we obtain that

$$f''(x) + g''(x) = 0 .$$

Since both $f''(x) \ge 0$ and $g''(x) \ge 0$, then $f''(x) = 0 = g''(x)$ and the result follows. □

Proof of Lemma 2. Observe that it is enough to consider h_I^* as given by

$$h_I^*(u) = -(u - r + 1)\psi(\theta_{r:u}) - (s - u)\psi(\theta_{(u+1):s}) , \quad u \in I ,$$

as the constant C does not depend on u. For any $u \in [c_{j-1}^*, c_j^*]$ define $t(u) = \frac{c_{j-1}^* - r + 1}{u - r + 1}$. Then we can write

$$\theta_{r:u} = t(u)\theta_{r:c_{j-1}^*} + (1 - t(u))\theta_j^* .$$

We now check each one of the condition of Lemma 2, beginning with (b). To prove that h_I^* is concave on the interval $[c_{j-1}^*, c_j^*]$, it is sufficient to show that $(u - r + 1)\psi(\theta_{r:u})$ and $(s - u)\psi(\theta_{(u+1):s})$ are convex on this interval. Take $g(u) = (u - r + 1)\psi(\theta_{r:u})$, and treat the vectors as column vectors. Then the first derivative of g is

$$\begin{aligned} g'(u) &= \psi(\theta_{r:u}) + (u - r + 1)t'(u)(\theta_{r:c_{j-1}^*} - \theta_j^*)^T \nabla\psi(\theta_{r:u}) \\ &= \psi(\theta_{r:u}) - t(u)(\theta_{r:c_{j-1}^*} - \theta_j^*)^T \nabla\psi(\theta_{r:u}) , \end{aligned}$$

where $\nabla\psi(\theta_{r:u})$ is the gradient of $\psi(\theta)$ evaluated at $\theta_{r:u}$ and the second equality follows from the fact that $(u-s+1)t'(u) = -t(u)$. The second derivative of g is then

$$\begin{aligned} g''(u) &= \overbrace{t'(u)(\theta_{r:c^*_{j-1}} - \theta^*_j)^T\nabla\psi(\theta_{r:u}) - t'(u)(\theta_{r:c^*_{j-1}} - \theta^*_j)^T\nabla\psi(\theta_{r:u})}^{=0} \\ &\quad + (-t'(u)t(u))(\theta_{r:c^*_{j-1}} - \theta^*_j)^T H\psi(\theta_{r:u})(\theta_{r:c^*_{j-1}} - \theta^*_j) \\ &\geq 0\,, \end{aligned}$$

because $H\psi(\theta_{r:u})$, the Hessian matrix of ψ evaluated at $\theta_{r:u}$, is positive definite and $-t'(u)t(u) \geq 0$. We conclude that g is convex on $[c^*_{j-1}, c^*_j]$. An analogous argument shows that $(s-u)\psi(\theta_{(u+1):s})$ is also convex, and then we finish verifying condition (b) in Lemma 1. Now we will show that, if h^*_I is constant on an interval $[c^*_{j-1}, c^*_j]$, then it must be equal to $h^*_I(s)$ on this interval. So suppose that h^*_I is constant on $[c^*_{j-1}, c^*_j]$. Then, for some α we must have that

$$-(u-r+1)\psi(\theta_{r:u}) - (s-u)\psi(\theta_{(u+1):s}) \;=\; \alpha \tag{2}$$

for all $u \in [c^*_{j-1}, c^*_j]$. Lemma 3 implies that $(u-r+1)\psi(\theta_{r:u})$ and $(s-u)\psi(\theta_{(u+1):s})$ must be linear functions on $[c^*_{j-1}, c^*_j]$, therefore $\psi(\theta_{r:u})$ and $\psi(\theta_{(u+1):s})$ are constants. Since ψ is strictly convex and $\theta_{r:u}$ (respectively $\theta_{(u+1):s}$) is a convex combination of $\theta_{r:c^*_{j-1}}$ and θ^*_j (respectively of $\theta_{c^*_j:s}$ and θ^*_j), we conclude that $\theta_{r:u} = \theta^*_j = \theta_{(u+1):s}$ for all $u \in [c^*_{j-1}, c^*_j]$. Moreover we have that

$$\theta_I \;=\; \frac{1}{|I|}\big[(u-r+1)\theta_{r:u} + (s-u)\theta_{(u+1):s}\big] \;=\; \theta^*_j$$

and therefore $h^*_I(u) = h^*_I(s)$, implying condition (c) in Lemma 1. We finish the proof by showing condition (a) in Lemma 1, that is that the minimum is attained at the interior of I. Suppose that this is not the case, that is that the minimum is attained at $h^*_I(s)$. Since $h^*_I(s)$ is also a maximum, because by definition

$$\frac{1}{n}l(r:u) + \frac{1}{n}l((u+1):s) \;\leq\; \frac{1}{n}l(r:s) \quad \text{for all } u \in I$$

we have that the function in the whole interval I must be constant. Following the same arguments as in the proof of condition (b) we conclude that $\theta^*_i = \theta^*_j$ for all $i, j \in \{1, \dots, k^*\}$, which is a contradiction with the hypothesis that $\theta^*_j \neq \theta^*_{j+1}$ for all $j = 1, \dots, k^*-1$. □

2.1 Bernoulli and Gaussian families of distributions

We present below two well-known examples of families of distributions where the hypotheses of Theorems 1 and 2 can be verified.

Example 1 (Bernoulli Random Variables)**.** *Let $A = \{a_1, \dots, a_d\}$ be a finite set and let*

$$\mathcal{F} = \Big\{p \in [0,1]^d \colon p(a_i) \geq 0 \text{ and } \sum_{i=1}^{d} p(a_i) = 1\Big\}$$

be the family of all probability distributions over A. Given $\mathbf{x} \in A^m$, the likelihood function can be written, for $C \in \mathcal{C}$ and $\theta = (p_1, \dots, p_k)$ as

$$\mathbb{P}_{(C,\theta)}(\mathbf{x}) = \prod_{j=1}^{k_C} \prod_{c=c_{j-1}+1}^{c_j} p_j(x_c)\,.$$

Example 2 (Gaussian Random Variables)**.** *Consider the family*

$$\mathcal{F} = \{f_{(\mu,\sigma^2)} \colon (\mu, \sigma^2) \in \mathbb{R} \times \mathbb{R}_+\} \tag{3}$$

of probability densities over $A = \mathbb{R}$, where $f_{(\mu,\sigma^2)}$ denotes the density of a Gaussian random variable with mean μ and variance σ^2. Fixing the change-point set C with $k_C = k$ and the vector of parameters $\theta = ((\mu_j, \sigma^2_j))_{j=1}^k$, the likelihood function assuming the variables in $\mathbf{x}$ are independent is given by

$$\mathbb{P}_{(C,\theta)}(\mathbf{x}) = \prod_{j=1}^{k_C} \prod_{c=c_{j-1}+1}^{c_j} (2\pi)^{-1/2}\sigma_j^{-1} \exp\Big\{-\frac{1}{2}\Big(\frac{x_c - \mu_j}{\sigma_j}\Big)^2\Big\}\,.$$

We now state two results that guarantee that both families of probability distributions given in Examples 1 and 2 satisfies Assumptions 1 and 2. Hence, both the dynamic programming and hierarchical segmentation algorithms provide consistent estimators of the change-point parameters (C^*, θ^*).

Proposition 1. *The family of discrete categorical distributions, as presented in Example 1, satisfies Assumptions 1 and 2.*

Proposition 2. *The family of Gaussian densities, as presented in Example 2, with unknown mean and constant known variance for all blocks satisfies Assumption 1 and 2.*

Proof of Proposition 1. Denote by $C^* = \{c^*_0, \dots, c^*_{k_{C^*}}\}$ and $p^* = (p^*_1, \dots, p^*_{k_{C^*}})$ the true change-point set and parameters, respectively. We will denote by $C = \{c_0, \dots, c_{k_C}\}$ and $p = (p_1, \dots, p_{k_C})$ any arbitrary change-point set and we will denote by I_j, respectively I^*_j, the interval between two change-points in C, respectively in C^*, that is $I_j = (c_{j-1}+1) : c_j$ and $I^*_j = (c^*_{j-1}+1) : c^*_j$. We begin by verifying hypotheses (PL1) and (PL2) in Assumption 1. In the case of categorical random variables, the log-likelihood function (3) for the sample $\mathbf{x}^n = \{\mathbf{x}^{(i)}\}_{i=1}^n$, evaluated at the maximum likelihood estimator for θ can be written as

$$l(C; \mathbf{x}^n) = \sum_{j=1}^{k_C} \sum_{a \in A} N_{I_j}(a) \log \frac{N_{I_j}(a)}{n|I_j|}\,, \tag{4}$$

where

$$N_{I_j}(a) \;=\; \sum_{i=1}^{n} \sum_{c=c_{j-1}+1}^{c_j} \mathbf{1}\{x^{(i)}_c = a\}\,. \tag{5}$$

To check (PL1) in Assumption 1 let any set $C \in \mathcal{C}$. By the Law of Large Numbers we have that

$$\frac{1}{n} l(C; \mathbf{x}^n) \;\to\; \sum_{j=1}^{k_C} |I_j| \sum_{a \in A} \hat{p}_{I_j}(a) \log \hat{p}_{I_j}(a) =: l^*(C)\,,$$

with

$$\hat{p}_{I_j}(a) \;=\; \sum_{r=1}^{k_{C^*}} \frac{|I_j \cap I^*_r|}{|I_j|} p^*_r(a) \qquad a \in A\,.$$

Moreover, if $C \not\supseteq C^*$ we have that

$$\begin{aligned} l^*(C) - l^*(C^*) \;&=\; \sum_{j=1}^{k_C} \sum_{r=1}^{k_{C^*}} |I_j \cap I^*_r| \sum_{a \in A} p^*_r(a) \log \hat{p}_{I_j}(a) - \sum_{r=1}^{k_{C^*}} |I^*_r| \sum_{a \in A} p^*_r(a) \log p^*_r(a) \\ &=\; \sum_{j=1}^{k_C} \sum_{r=1}^{k_{C^*}} |I_j \cap I^*_r| \sum_{a \in A} p^*_r(a) \log \frac{\hat{p}_{I_j}(a)}{p^*_r(a)} \\ &<\; 0 \end{aligned} \tag{6}$$

unless $\hat{p}_{I_j} = p^*_r$ for all r and j with $|I_j \cap I^*_r| \neq \emptyset$. But this can only happen if $C \supseteq C^*$, which is a contradiction. To verify hypothesis (PL2) observe that for any $C \supseteq C^*$

$$\begin{aligned} l(C; \mathbf{x}^n) - l(C^*; \mathbf{x}^n) \;&=\; \sum_{j=1}^{k_C} \sum_{a \in A} N_{I_j}(a) \log \frac{N_{I_j}(a)}{n|I_j|} - \sum_{j=1}^{k_{C^*}} \sum_{a \in A} N_{I^*_j}(a) \log \frac{N_{I^*_j}(a)}{n|I^*_j|} \\ &\leq\; \sum_{j=1}^{k_C} \sum_{a \in A} N_{I_j}(a) \log \frac{N_{I_j}(a)}{n|I_j|} - \sum_{j=1}^{k_{C^*}} \sum_{a \in A} N_{I^*_j}(a) \log p^*_j(a)\,, \end{aligned}$$

where the last inequality follows because $N_{I^*_j}(a)/n|I^*_j|$ are the maximum likelihood estimators for $p^*_j(a)$. As $C \supseteq C^*$, we have that any interval I^*_r contains one or more intervals I_j, and

$$N_{I^*_r}(a) \;=\; \sum_{j : I_j \subseteq I^*_r} N_{I_j}(a) \qquad \text{for all } a \in A\,.$$

Then, we can write the difference above as

$$\sum_{j=1}^{k_C} \sum_{a \in A} N_{I_j}(a) \log \frac{N_{I_j}(a)/n|I_j|}{p^*_{i_j}(a)}$$

where i_j is the corresponding index in C^*. Therefore we obtain that

$$l(C;\mathbf{x}^n) - l(C^*;\mathbf{x}^n) \;\leq\; \sum_{j=1}^{k_C} n|I_j|\, D(\widehat{p}_j; p^*_{i_j})\,,$$

where

$$\widehat{p}_j(a) \;=\; \frac{N_{I_j}(a)}{n|I_j|}\,, \quad a \in A$$

and D denotes the *Kullback-Leibler* divergence between the probability distributions $\{\widehat{p}_j(a)\}_{a\in A}$ and $\{p^*_{i_j}(a)\}_{a\in A}$. By a well-known inequality, see for example [2, Lemma 6.3] we have that

$$D(\widehat{p}_j(a)|p^*_{i_j}(a)) \;\leq\; \sum_{a\in A} \frac{|\widehat{p}_j(a) - p^*_{i_j}(a)|^2}{p^*_{i_j}(a)}\,.$$

As the difference $N_{I_j}(a) - n|I_j|p_j(a)$ can be written as a sum of zero-mean independent random variables with finite variance, we have, by the Law of the Iterated Logarithm, see for example [1, Theorem 3.52], that

$$|\widehat{p}_j(a) - p^*_{i_j}(a)| \;\leq\; \sqrt{\frac{c\log\log n|I_j|}{n|I_j|}} \;\leq\; \sqrt{\frac{c\log\log nm}{n|I_j|}}$$

for a given constant c and for all $a \in A$, eventually almost surely as $n \to \infty$. Hence for all $\delta > 0$ we have that

$$|\widehat{p}_j(a) - p_{i_j}(a)| \;\leq\; \sqrt{\frac{\delta\log n}{n|I_j|}}$$

eventually almost surely as $n \to \infty$. Finally, we obtain that

$$l(C;\mathbf{x}^n) - l(C^*;\mathbf{x}^n) \;\leq\; \frac{k_C\delta}{p^*_{\min}}\log n \;\leq\; \frac{m\delta}{p^*_{\min}}\log n\,,$$

with $p^*_{\min} = \min\{p^*_j(a)\colon p^*_j(a) > 0, j = 1,\ldots,k_{C^*}, a \in A\}$. On the other hand, it is easy to see that

$$l(C;\mathbf{x}^n) - l(C^*;\mathbf{x}^n) \;\geq\; 0$$

for any $C \supseteq C^*$. Then, hypothesis (PL2) holds for $v(n) = \delta\log n$, for any $\delta > 0$. This establishes the conditions on Assumption 1.

Now we verify the hypotheses (H1)-(H2) in Assumption 2. Observe that as in the case of (PL1), the Law of Large Numbers can be invoked to prove that

$$\frac{1}{n}l(I;\mathbf{x}^n) \;\to\; l^*(I)\colon = |I|\sum_{a\in A}\hat{p}_I(a)\log\hat{p}_I(a) \tag{7}$$

for any integer interval $I \in \mathcal{I}$. Now, for $I = r:s$ consider the function $h^*_I : I \to \mathbb{R}$ defined as

$$h^*(u) \;=\; -l^*(r:u) - l^*((u+1):s)\,, \quad u \,\in\, I\,.$$

As $l^*(I)$ satisfies the hypotheses of Lemma 2 with $\psi(\hat{p}) = \sum_{a\in A}\hat{p}(a)\log\hat{p}(a)$ we have that

$$\min_{u\in I\setminus\{s\}\cap C^*} h^*(u) \;<\; \min_{u\notin I\setminus\{s\}\cap C^*} h^*(u)$$

whenever $I\setminus\{s\}\cap C^* \neq \emptyset$, concluding the proof of (H1). The proof of (H2) follows by (PL2) in Assumption 2. □

Proof of Proposition 2. For the Gaussian distribution, the maximum likelihood estimator of the mean on an interval I is given by

$$\widehat{\mu}_I \;=\; \frac{1}{n|I|}\sum_{c\in I}\sum_{i=1}^{n} x_c^{(i)}\,.$$

Then the log-likelihood function defined in the main document by (2.4) for a change point set $C = (c_0,\ldots,c_k) \in \mathcal{C}$ is given by

$$l(C;\mathbf{x}^n) = -\frac{nm}{2}\log(2\pi\sigma_*^2) - \frac{1}{2\sigma_*^2}\sum_{j=1}^{k_C}\sum_{c\in I_j}\sum_{i=1}^{n}(x_c^{(i)} - \widehat{\mu}_{I_j})^2\,,$$

where $I_j = (c_{j-1}+1) : c_j$ for $j = 1,\ldots,k$. Notice that by the Law of Large Numbers, for all $j = 1,\ldots,k$ we have that

$$\widehat{\mu}_{I_j} \;=\; \sum_{r=1}^{k_{C^*}} \frac{|I_j\cap I_r^*|}{|I_j|}\left[\frac{1}{n|I_j\cap I_r^*|}\sum_{c\in I_j\cap I_r^*}\sum_{i=1}^{n} x_c^{(i)}\right] \;\xrightarrow{a.s.}\; \sum_{r=1}^{k_{C^*}}\frac{|I_j\cap I_r^*|}{|I_j|}\mu_r^* \;=:\; \bar{\mu}_{I_j}\,.$$

Observe also that

$$\begin{aligned}\sum_{j=1}^{k_C}\sum_{c\in I_j}\sum_{i=1}^{n}(x_c^{(i)} - \widehat{\mu}_{I_j})^2 \;&=\; \sum_{j=1}^{k_C}\sum_{r=1}^{k_{C^*}}\sum_{c\in I_j\cap I_r^*}\sum_{i=1}^{n}(x_c^{(i)} - \mu_r^* + \mu_r^* - \widehat{\mu}_{I_j})^2\\ &=\; \sum_{j=1}^{k_C}\sum_{r=1}^{k_{C^*}}\sum_{c\in I_j\cap I_r^*}\sum_{i=1}^{n}(x_c^{(i)} - \mu_r^*)^2 + 2(x_c^{(i)} - \mu_r^*)(\mu_r^* - \widehat{\mu}_{I_j})\\ &\qquad + (\mu_r^* - \widehat{\mu}_{I_j})^2\,.\end{aligned}$$

Then, as $n\to\infty$ we have that

$$\frac{1}{n}\sum_{j=1}^{k_C}\sum_{r=1}^{k_{C^*}}\sum_{c\in I_j\cap I_r^*}\sum_{i=1}^{n}(x_c^{(i)} - \mu_r^*)^2 \;\xrightarrow{a.s.}\; \sum_{j=1}^{k_C}\sum_{r=1}^{k_{C^*}}|I_j\cap I_r^*|\sigma_*^2 \;=\; m\sigma_*^2\,.$$

In the same way

$$\frac{1}{n}\sum_{j=1}^{k_C}\sum_{r=1}^{k_{C^*}}\sum_{c\in I_j\cap I_r^*}\sum_{i=1}^{n}2(x_c^{(i)} - \mu_r^*)(\mu_r^* - \widehat{\mu}_{I_j}) \;\xrightarrow{a.s.}\; 0$$

and

$$\frac{1}{n}\sum_{j=1}^{k_{C^*}}\sum_{r=1}^{k_{C^*}}\sum_{c\in I_j\cap I_r^*}\sum_{i=1}^{n}(\mu_r^* - \widehat{\mu}_{I_j})^2 \;\xrightarrow{a.s.}\; \sum_{j=1}^{k_C}\sum_{r=1}^{k_{C^*}}|I_j\cap I_r^*|(\mu_r^* - \bar{\mu}_{I_j})^2\,.$$

Then, for any $C\in\mathcal{C}$, by some manipulation we obtain that the almost sure limit of the maximum log-likelihood function is

$$\frac{1}{n}l(C;\mathbf{x}) \;\xrightarrow{a.s.}\; -\frac{m}{2}\log(2\pi\sigma_*^2) - \frac{1}{2\sigma_*^2}\left[m\sigma_*^2 + \sum_{j=1}^{k_C}\sum_{r=1}^{k_{C^*}}|I_j\cap I_r^*|(\mu_r^* - \bar{\mu}_{I_j})^2\right] \;=:\; l^*(C)\,.$$

We begin by verifying conditions (PL1) and (PL2) on Assumption 1. Observe that for any interval $I\in\mathcal{I}$ we have that

$$\begin{aligned}\sum_{r=1}^{k_{C^*}}|I\cap I_r^*|\,(\mu_r^* - \bar{\mu}_I)^2 \;&=\; \sum_{r=1}^{k_{C^*}}|I\cap I_r^*|\,\mu_r^{*,2} - 2\bar{\mu}_I\sum_{r=1}^{k_{C^*}}|I\cap I_r^*|\,\mu_r^* + |I|\,\bar{\mu}_I^2\\ &=\; \sum_{r=1}^{k_{C^*}}|I\cap I_r^*|\mu_r^{*,2} - |I|\,\bar{\mu}_I^2\,.\end{aligned}\tag{8}$$

Then the function $l^*(C)$ is given by

$$l^*(C) \;=\; -\frac{m}{2}[\log(2\pi\sigma_*^2)+1] - \sum_{r=1}^{k_{C^*}}|I_r^*|\mu_r^{*,2} + \sum_{j=1}^{k_C}|I_j|\bar{\mu}_{I_j}^2\,.$$

By the strict convexity of $x\mapsto x^2$ we have that

$$\sum_{j=1}^{k_C}|I_j|\bar{\mu}_{I_j}^2 \;\le\; \sum_{j=1}^{k_C}|I_j|\sum_{r=1}^{k_{C^*}}\frac{|I_j\cap I_r^*|}{|I_j|}\mu_r^{*,2} \;=\; \sum_{r=1}^{k_{C^*}}|I_r^*|\mu_r^{*,2}$$

then

$$l^*(C) \;\leq\; l^*(C^*)\,.$$

Moreover, the inequality is strict unless $C \supseteq C^*$, and then (PL1) follows.

To prove (PL2) in Assumption 1, if we define the empirical variance of an interval I as

$$\hat{\sigma}_I^2 = \frac{1}{n|I_j|}\sum_{c\,\in\, I_j}\sum_{i=1}^{n}(X_c^{(i)} - \hat{\mu}_j)^2$$

then the log-likelihood for any block over a finite sample can be written as

$$l(C;\mathbf{x}^n) = -\frac{nm}{2}\log(\sigma_*^2) - \frac{1}{2\sigma_*^2}\sum_{r=1}^{k_C}\hat{\sigma}_{I_r}^2$$

Given a change point set C, define $\mathcal{I}_r(C) = \{j \in (1:k_C) | I_j \subseteq I_r^*\}$, the set of all indices whose blocks defined by C are contained in I_r^*. If C does not segment I_r^*, then there is only one index in $\mathcal{I}_r$ and the set that it indexes is exactly I_r^*. The log-likelihood difference to the true change point set is then

$$l(C;\mathbf{x}^n) - l(C^*;\mathbf{x}^n) = \frac{n}{2\sigma_*^2}\sum_{r=1}^{k_{C^*}}\left[|I_r{}^*|\hat{\sigma}_{I_r^*}^2 - \sum_{j\,\in\,\mathcal{I}_r(C)}|I_j|\hat{\sigma}_{I_j}^2\right] \quad .$$

The empirical variance estimator for the whole block satisfies

$$\begin{aligned}
\hat{\sigma}_{I_r^*}^2 &= \frac{1}{n|I_r|}\sum_{c\,\in\, I_r^*}\sum_{i=1}^{n}(X_c^{(i)} - \hat{\mu}_{I_r^*})^2 \\
&= \frac{1}{n|I_r|}\sum_{c\,\in\, I_r^*}\sum_{i=1}^{n}\left[\left(X_c^{(i)} - \mu_r^*\right)^2\right] - (\hat{\mu}_{I_r^*} - \mu_r^*)^2 \\
&\leq \frac{1}{n|I_r|}\sum_{c\,\in\, I_r^*}\sum_{i=1}^{n}\left[\left(X_c^{(i)} - \mu_r^*\right)^2\right]\,.
\end{aligned}$$

A similar calculation for the variance of the nested blocks yields

$$\begin{aligned}
\hat{\sigma}_{I_j}^2 &= \frac{1}{n|I_j|}\sum_{c\,\in\, I_j}\sum_{i=1}^{n}(X_c^{(i)} - \hat{\mu}_{I_j})^2 \\
&= \frac{1}{n|I_j|}\sum_{c\,\in\, I_j}\sum_{i=1}^{n}\left[\left(X_c^{(i)} - \mu_r^*\right)^2\right] - (\hat{\mu}_{I_j} - \mu_r^*)^2\,.
\end{aligned}$$

Define $j' = \underset{\mathcal{I}_r(C)}{\operatorname{argmax}}(\hat{\mu}_j - \mu_r^*)^2$, we have

$$\begin{aligned}
\frac{n}{2\sigma_*^2}\left(|I_r^*|\hat{\sigma}_r^2 - \sum_{j\,\in\,\mathcal{I}_r(C)}|I_j|\hat{\sigma}_j^2\right) &= \frac{n}{2\sigma_*^2}\left(\sum_{j\,\in\,\mathcal{I}_r(C)}(\hat{\mu}_j - \mu_r^*)^2\right) \\
&\leq \frac{|I_r^*|n}{2\sigma_*^2}(\hat{\mu}_{j'} - \mu_r^*)^2 \\
&= \frac{|I_r^*|}{2|I_{j'}|^2\sigma_*^2}\left[\frac{1}{\sqrt{n}}\sum_{c\,\in\, I_{j'}}\sum_{i=1}^{n}\left(X_c^{(i)} - \mu_r^*\right)\right]^2\,.
\end{aligned}$$

Define

$$S_{j^*} = \left[\frac{1}{\sqrt{n\log\log n}}\sum_{c\,\in\, I_{j'}}\sum_{i=1}^{n}\left(X_c^{(i)} - \mu_r^*\right)\right]\,.$$

By the law of the iterated logarithm, there exists $\epsilon_{j^*} > 1$ such that

$$\limsup_{n\to\infty} S_{j^*} < \epsilon_{j^*}\,.$$

By lim sup properties,

$$\limsup_{n\to\infty} S_{j^*}^2 < \epsilon_{j^*}^2 .$$

Dividing the terms inside each block by $\log\log n$, we have

$$\begin{aligned}\limsup_{n\to\infty} \frac{|I_r^*|}{2|I_{j^*}|^2\sigma_*^2} S_{j^*}^2 &\leq \frac{|I_r^*|}{2|I_{j^*}|^2\sigma_*^2} \limsup_{n\to\infty} \left(S_{j^*}^2\right) \\ &\leq \frac{|I_r^*|}{2|I_{j^*}|^2\sigma_*^2} \epsilon_j^2 .\end{aligned}$$

Finally

$$\begin{aligned}\frac{1}{\log\log n} l(C;\mathbf{X}) - l(C^*;\mathbf{X}) &\leq \limsup_{n\to\infty} \frac{1}{\log\log n} l(C;\mathbf{X}) - l(C^*;\mathbf{X}) \\ &\leq \sum_{r=1}^{k_{C^*}} \frac{|I_r^*|}{2|I_{j^*}|^2\sigma_*^2} \epsilon_j^2 \\ &\leq \epsilon ,\end{aligned}$$

for some $\epsilon > 0$. This establishes condition (PL2).

We now verify conditions (H1) and (H2) on Assumption 2. Observe that for any interval $I = r : s \in \mathcal{I}$ we have that

$$\begin{aligned}\sum_{r=1}^{k_{C^*}} |I \cap I_r^*| \left(\mu_r^* - \bar{\mu}_I\right)^2 &= \sum_{r=1}^{k_{C^*}} |I \cap I_r^*| \, \mu_r^{*,2} - 2\bar{\mu}_I \sum_{r=1}^{k_{C^*}} |I \cap I_r^*| \, \mu_r^* + |I| \, \bar{\mu}_I^2 \\ &= \sum_{r=1}^{k_{C^*}} |I \cap I_r^*| \mu_r^{*,2} - |I| \, \bar{\mu}_I^2 .\end{aligned}$$

Then the function $h_I^*(u)$ in (4.2) is given by

$$h_I^*(u) = \alpha(I, \theta^*) + (u - r + 1)\psi(\bar{\mu}_{r:u}) + (s - u)\psi(\bar{\mu}_{(u+1):s}) ,$$

with $\psi(\mu) = \mu^2$, $\mu \in \mathbb{R}$. As h_I^* satisfies the conditions of Lemma 2 we have that (H1) follows. The proof of (H2) in Assumption 2 is obtained in the same way as (PL2) in Assumption 1. □

2.2 Computational complexity of hierarchical segmentation algorithm

Proposition 3. *Assume that the data is distributed from the proposed statistical model, and that the latter satisfies the consistency conditions for Theorem 2. Then, the hierarchical segmentation algorithm will asymptotically perform exactly $2k_{C^*} - 1$ recursive calls. Moreover, its asymptotic complexity is of order $O(T(n, m) + mk_{C^*})$, where $T(n, m)$ is the time complexity to compute the sufficient statistics for each interval.*

Proof. We will first prove that the algorithm will eventually almost surely do exactly $2k_{C^*} - 1$ recursive calls by induction on k_{C^*}. Consider the base case $C^* = \{0, m\}$, when there is no change-point except the extremes. The first run of the algorithm is always on $1 : m$. Since it is asymptotically correct, it halts after the first iteration. Therefore, the number of recursive calls is $1 = 2k_{C^*} - 1$. Now suppose that the formula holds for $k_{C^*} \leq K - 1$ for any value of m, we will prove that it holds for $k_{C^*} = K$. At the first call, the algorithm selects a change-point c and creates a recursive call on $1 : c$ and on $(c + 1) : m$. Let N_l be the number of change-points on $1 : c$ and N_r be the number of change-points on $(c + 1) : m$ Using induction hypothesis, we have that the algorithm does $2(N_l + 1) - 1$ calls on $1 : c$ and $2(N_r + 1) - 1$ on $(c + 1) : m$. Notice that $N_l + N_r = K - 2$ because $k_{C^*} = K$ implies there are $K - 1$ change-points for the original change-point set, but c is not counted on N_l or N_r. Hence, the total number of calls is

$$1 + (2N_l + 1) + (2N_r + 1) = 2(N_l + N_r) + 3 = 2K - 1 .$$

To obtain the final complexity, a call in the interval $r : s$ the algorithm does $|s - r + 1| \leq m + 1$ comparisons and memory checks, and therefore has complexity of $O(mk_{C^*})$). □

3 Simulations for Gaussian random variables

We provide the results of the simulations in the case of normal random variables.

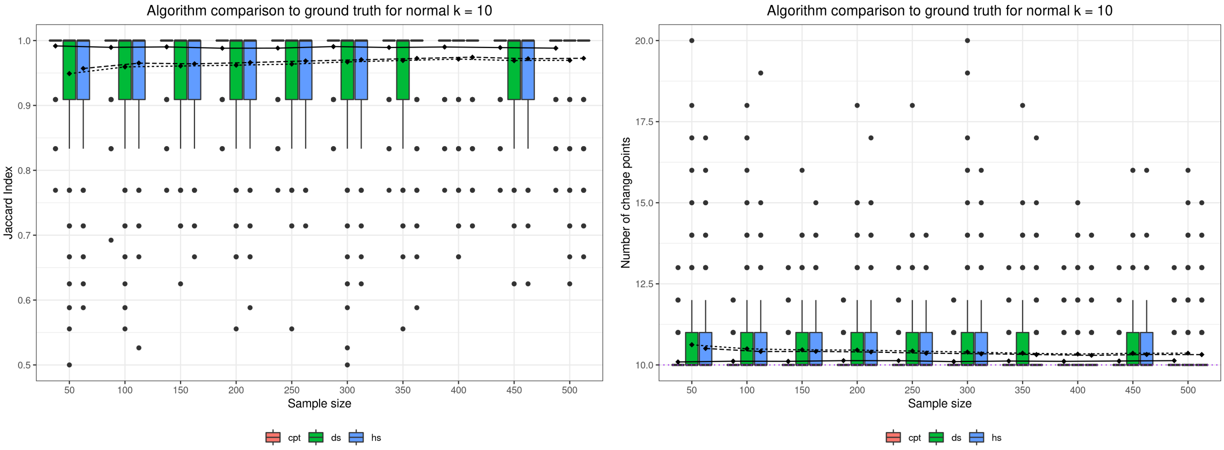

Figure 1: Comparison of estimated change point sets between algorithms and against ground truth for the Gaussian case using the Jaccard Index and estimated number of change-points for $k^* = 10$

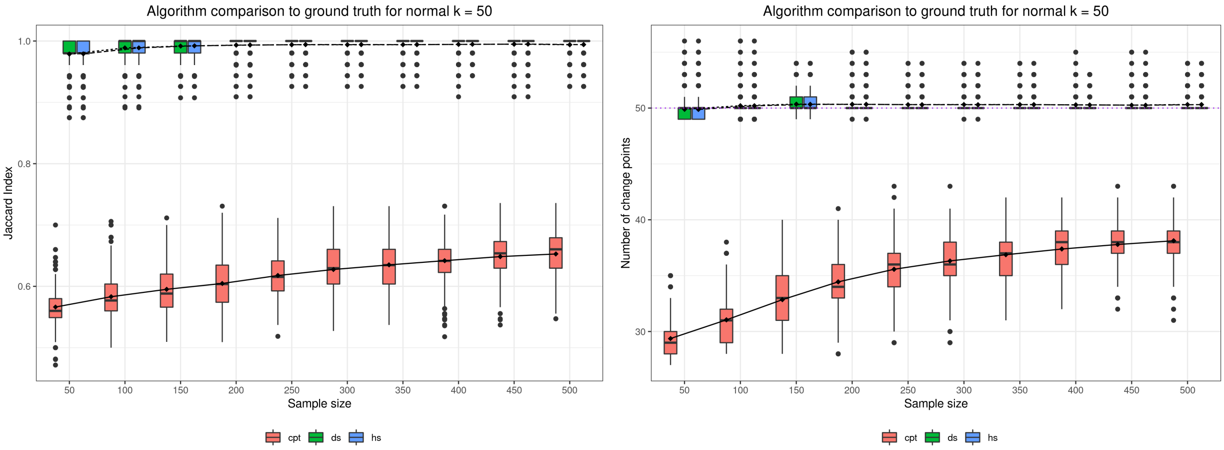

Figure 2: Comparison of estimated change point sets between algorithms and against ground truth for the Gaussian case using the Jaccard Index and estimated number of change-points for $k^* = 50$

4 ROH islands detection

4.1 Comparison with PLINK, 99% quantile

As discussed in the paper, we also considered a 99% quantile cutoff for PLINK and `blockcpd`, for African and European populations. The results are shown in Figure 3.

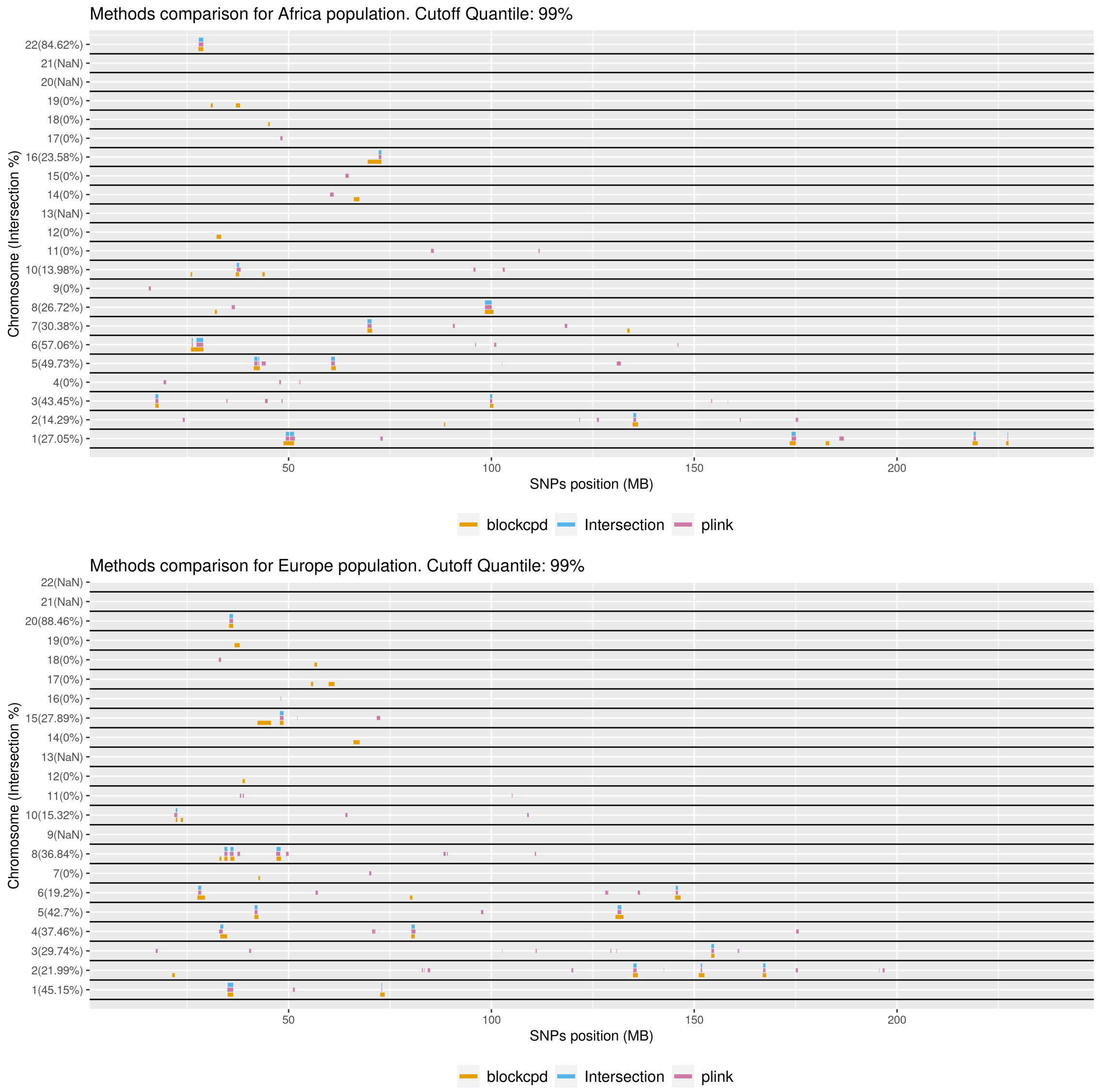

Figure 3: Comparison of the detected ROH islands across the genome by our hierarchical algorithm (`blockcpd`) and PLINK. The ROH islands detected by both methods are shown for African (top) and European (bottom) populations, together with their intersection. The intersection percentage is displayed on the vertical axis.

4.2 Full genome sequences

The `blockcpd` algorithm was also applied to full genome sequences from the HGDP database. The results comparing the method to PLINK and the method by Pemberton et al (2012) are shown in Figure 4.

As in the main article, we considered a quantile cutoff of 95%. For PLINK, the cutoff is based on the frequency distribution that SNPs appear in ROH. For `blockcpd`, the cutoff is based on the probability parameter of the blocks.

For the comparison to Pemberton et al (2012) method, we first identified the ROH using the same three classes as defined in the original method. Then, instead of comparing our method to each of the three probabilities obtained by their method, we compared our method to the maximum of the three probabilities per SNP. Hence, we called ROH islands on Pemberton et al (2012) method by performing a quantile cutoff on the maximum of the three probabilities, which are represented by the blocks on Figure 4.

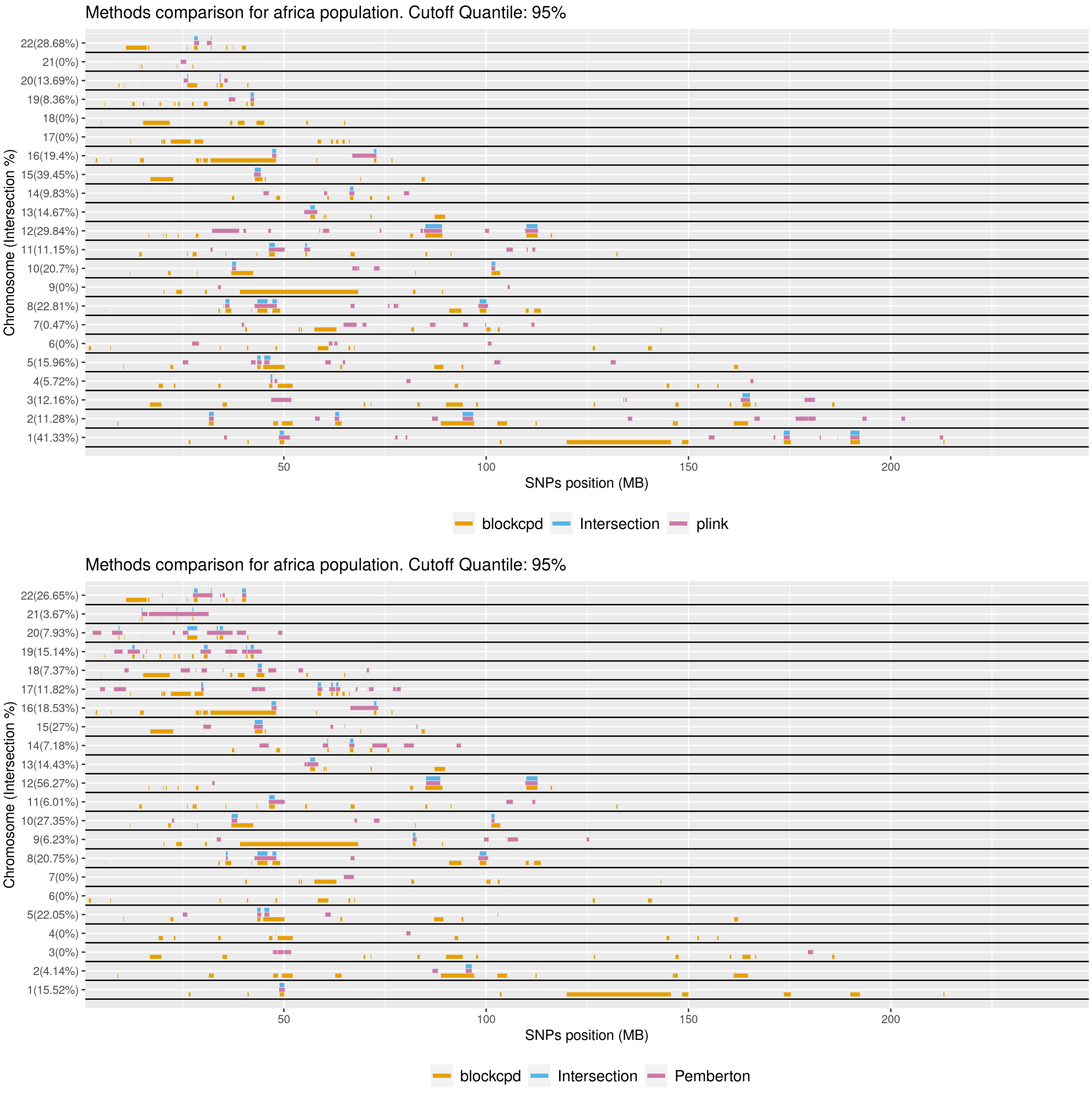

Figure 4: Analysis of full genome sequences from HGDP database, for African population, and comparison with PLINK (top) and the method by Pemberton et al (2012) (bottom).

References

[1] Leo Breiman. *Probability and stochastic processes; with a view toward applications*. Houghton Mifflin Co, 1969.

[2] I. Csiszar and Z. Talata. Context tree estimation for not necessarily finite memory processes, via bic and mdl. *IEEE Transactions on Information Theory*, 52(3):1007–1016, 2006.
